# Treatment of Hemorrhoids by Iodoform in Ether

**Published:** 1895-03

**Authors:** William J. Cox

**Affiliations:** Flovilla, Ga.


### CORRESPONDENCE.

### TREATMENT OF HEMORRHOIDS BY IODOFORM IN ETHER.

FLOVILLA, GA., February, 1895.

The writer having a large number of patients suffering with hemorrhoids, and most of them needing radical measures, was induced to use injections of carbolic acid, and with a marked degree of success; but it did not meet the requirements in every particular, and then there is slight danger of embolism from its use. This danger has, however, been overestimated by those who do not know how to use it, and possibly, also, by those prejudiced against its use.

After some reasoning along on this line, and after seeing the beautiful results of iodoform dissolved in ether in cysts, goitre, and also in hydrocele, I was induced to give it a trial in hemorrhoids. This I find to be the most satisfactory of all injections yet used. The *modus operandi* is very simple. After thoroughly emptying the bowels with some purgative, give a large enema. When patient is ready for operation, I find the knee-chest position an excellent one for the procedure. A small injection of some antiseptic is then given. I use boric acid twenty grains to the ounce, after which a small syringeful of a four per cent. solution of cocaine is injected into the rectum. A long speculum is then introduced, through which I pass a piece of antiseptic gauze. Each tumor is then exposed and a small amount, about ten drops, of a saturated solution of iodoform in ether is injected into the base of each tumor; if very large on each side of it. The patient is then given a suppository of cocaine and opium to be used at intervals, as indicated. Opium and bismuth are given to produce constipation; after three or four days an injection of castor oil is given, or preferably olive oil, and also a dose internally. The bowels are kept loose for several days, when the patient will be well. I never stop patients from their avocation. There is usually no sloughing or any bad symptom whatever, nor very much pain, and the fact that the patients can go about their daily employment makes it quite the favorite plan for me. While I claim no originality for this, I do not know of it having been used before.

I want it distinctly understood that this solution is not injected into the tumor as the carbolic injection, but in the tissue beneath the growth.

I have used it in about a dozen cases with complete success. I hope the profession will give it a trial and report results.

WILLIAM J. COX, M.D.

---

### TEST FOR ALBUMIN IN URINE.

Dr. McCourt (*Medical Record*, January 12) says:

During several years past I have employed, for myself and a number of my professional friends, a modification of Mehn's test, as modified by Millard, consisting of

℞. Acid. phenic. glac., ninety-five per cent . . . . ℥ ss.

Acid. acetic. puri . . . . . . . . . . . . . . . . ℥ j. ʒ vj.

Add liquor potassæ . . . . . . . . . . . . . . . ℥ v. ʒ iv.

The glacial phenic acid should be reduced to the ninety-five per cent. solution at the moment of compounding, else the resultant fluid will be turbid; and all ingredients must be chemically pure.

To 5 c.c. of urine in a test-tube add 0.5 c.c. of the test, slowly from a pipette, to underlie the urine. In the presence of albumin a white, milky ring, of varying depth and density, to a faint milky cloud, will give the qualitative and quantitative result.

This is an ideal test for albumin, so accurate, sensitive, and convenient as to meet every requirement. The test will react with 1 to 300,000 of artificial albumin and water, and is not obscured by peptones, phosphates, urates, nitrate of urea, nor even by sugar. These merits are not possessed by any other test for albumin with which I am acquainted; and I am familiar with all. The exposure from frequent use will in time change its color from clear to light brown; but this change of color does not impair its delicacy. Having traversed this entire field, and tested nearly all the tests known to us in urinalysis, I have come to rely wholly on this one for albumin.

